# Bipolar Analog Memristors as Artificial Synapses for Neuromorphic Computing

**DOI:** 10.3390/ma11112102

**Published:** 2018-10-26

**Authors:** Rui Wang, Tuo Shi, Xumeng Zhang, Wei Wang, Jinsong Wei, Jian Lu, Xiaolong Zhao, Zuheng Wu, Rongrong Cao, Shibing Long, Qi Liu, Ming Liu

**Affiliations:** 1 Institute of Microelectronics of Chinese Academy of Sciences, Beijing 100029, China; wangrui@ime.ac.cn (R.W.); zhangxumeng@ime.ac.cn (Xr.Z.); wangwei_esss@nudt.edu.cn (W.W.); weijinsong@ime.ac.cn (J.W.); lujian@ime.ac.cn (J.L.); zhaoxiaolong@ime.ac.cn (X.Z.); wuzuheng@ime.ac.cn (Z.W.), caorongrong@ime.ac.cn (R.C.); liuming@ime.ac.cn (M.L.); 2 University of Chinese Academy of Sciences, Beijing 100049, China; 3 University of Science and Technology of China, Hefei 230026, China; longshibing@ime.ac.cn

**Keywords:** memristor, artificial synapse, neuromorphic computing

## Abstract

Synaptic devices with bipolar analog resistive switching behavior are the building blocks for memristor-based neuromorphic computing. In this work, a fully complementary metal-oxide semiconductor (CMOS)-compatible, forming-free, and non-filamentary memristive device (Pd/Al_2_O_3_/TaO_x_/Ta) with bipolar analog switching behavior is reported as an artificial synapse for neuromorphic computing. Synaptic functions, including long-term potentiation/depression, paired-pulse facilitation (PPF), and spike-timing-dependent plasticity (STDP), are implemented based on this device; the switching energy is around 50 pJ per spike. Furthermore, for applications in artificial neural networks (ANN), determined target conductance states with little deviation (<1%) can be obtained with random initial states. However, the device shows non-linear conductance change characteristics, and a nearly linear conductance change behavior is obtained by optimizing the training scheme. Based on these results, the device is a promising emulator for biology synapses, which could be of great benefit to memristor-based neuromorphic computing.

## 1. Introduction

Over the last decades, rapid advances in digital computing system based on complementary metal-oxide semiconductor (CMOS) integrated circuit technology have substantially changed society. However, due to the limitations of classical von-Neumann computers (the von-Neumann bottleneck) in speed, power efficiency, and parallel processing, there are urgent demands for novel computing structures and systems [1]. The human brain is likely to be the most efficient computing system, because the operating frequency of our brain is in the range of 1–10 Hz, and it consumes only around 1–10 W of power, which means the energy consumption per synaptic event is only approximately 1–100 fJ [2]. Therefore, the novel computing system—neuromorphic computing, inspired by the brain—has attracted scientists’ attention in recent years for its advantages, such as being massively parallel and fault-tolerant. The weight modulation ability of synapses is known as synaptic plasticity, which is believed to be the primary reason for learning and memory in the brain. In order to implement neuromorphic computing, such as artificial neural networks (ANN), an electronic synaptic device is necessary. 

Recently, the implementation of artificial synapses with memristors has been proposed. Memristors are two compact terminal devices that change their resistances when subjected to electrical stimulation [3,4,5,6]. Several memristors, ranging from resistive random access memory (RRAM) [7,8,9,10,11], to phase change memory (PCM) [12], to ferroelectric RAM [13,14,15], have been proposed for neuromorphic computing applications as artificial synapses. Several memristors based on new materials [16,17] have been proposed for neuromorphic computing. However, when memristors are employed in neuromorphic computing systems (e.g., artificial neuron networks), binary memristors with only two resistance states (i.e., high resistance state (HRS) and low resistance state (LRS)) have been proven to be effective only in some specific applications [18,19]. In some neuromorphic computing systems designed for complex applications, such as image recognition, the use of only two states as synaptic weights presents disadvantages in performances [20,21]—for example, low accuracy or area-efficiency. On the other hand, in biology neuromorphic systems, synaptic weights are continuously tunable in depression and potentiation; thus, memristors with gradually changing conductance in bipolarity could be more like the biology synapse, and can therefore emulate brain functions better than binary memristors. As artificial synapses, memristors with tunable conductance have attracted growing attention for being promising candidates for weight storage in neuromorphic computing systems, owing to the advantages in accuracy and area-efficiency. Several methods have been discussed to implement analog-resistive switching behavior, including using multiple memristors to construct one synapse [22], utilizing a unipolar analog behavior in some metal oxide-based filamentary memristors [11,23,24], optimizing programming schemes [25,26], adding heat enhancement layers [27], or using non-filamentary memristors [28,29,30]. Compared with the filamentary memristors, non-filamentary memristors can implement multilevel states more easily, but usually have poorer retention [31,32,33] However, realizing bipolar analog conductance change in both SET (transition from HRS to LRS) and RESET (transition from LRS to HRS) processes with satisfying retention time remains an open challenge. 

In this paper, a fully CMOS-compatible, forming-free, and non-filamentary memristor device based on Ta/TaO_x_/Al_2_O_3_/Pd, with analog SET and RESET processes, is proposed for neuromorphic computing as an artificial synapse. The direct current (DC) sweeping results demonstrate that the device has bipolar analog resistance switching behavior, and the multilevel conductance states can be obtained with satisfying retention time. Synaptic plasticity, including long-term potentiation/depression (LTP/LTD), paired-pulse facilitation (PPF), and spiking-time-dependent plasticity (STDP), can be mimicked by our devices. For the applications in ANN, determined target conductance states and the linearity of conductance change are carefully examined.

## 2. Materials and Methods 

**Figure 1:**
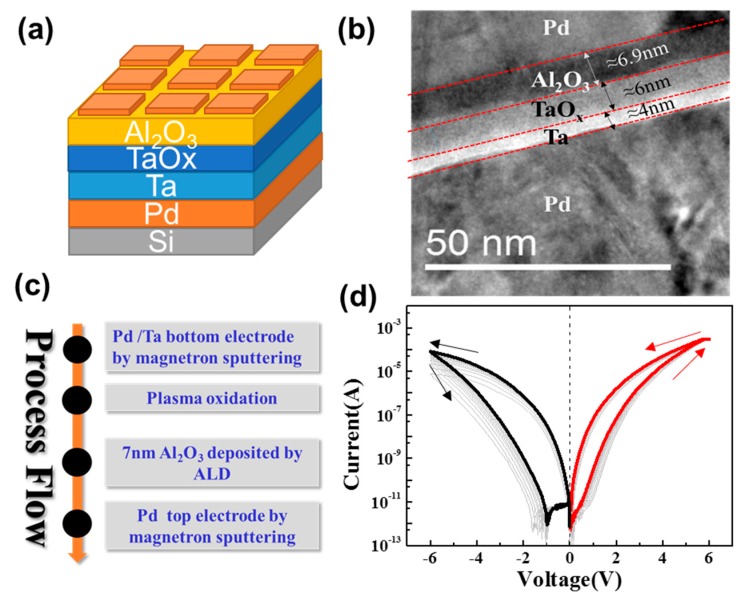
(**a**) The schematic, (**b**) a cross sectional transmission electron microscopy (TEM) image of the Ta/TaO_x_/Al_2_O_3_/Pd device, (**c**) the fabrication processes of the Ta/TaO_x_/Al_2_O_3_/Pd device, and (**d**) typical *I–V* curves of the Ta/TaO_x_/Al_2_O_3_/Pd showing bipolar analog switching (SET voltage = 6 V, RESET voltage = −6 V).

The metal–insulator (double functional layer)–metal structure and the cross-sectional transmission electron microscopy (TEM) image of the Ta/TaO_x_/Al_2_O_3_/Pd device are shown in Figure 1a,b, respectively. The fabrication process of the device is shown in Figure 1c. First, the Si substrate was cleaned with acetone, ethanol, and de-ionized water. 30 nm-thick Pd and 15 nm-thick Ta as the bottom electrode were deposited on the Si substrate by magnetron sputtering. A TaO_x_ layer was formed by rapid thermal annealing (RTA) carried out for 300 s in plasma O_2_ by plasma-enhanced chemical vapor deposition (PECVD) at 275 °C. Direct oxygen plasma with a power of 100 W was applied on the Ta film. After RTA, 7 nm-thick Al_2_O_3_ was deposited by atom layer deposition (ALD). Finally, 40 nm Pd as the top electrode was deposited by magnetron sputtering after the lithography process. For our device, the highest temperature of the process is only 275 °C (below 400 °C), and all the materials (Pd, Ta, Al) were CMOS compatible. As a result, our device was fully CMOS compatible.

The DC electrical characteristics of the device were measured by an Agilent B1500A semiconductor parameter analyzer (Santa Rosa, CA, US). During the electrical measurement, the voltage was applied to the top Pd electrode, while the Ta/Pd bottom electrode was tied to ground. 

## 3. Results and Discussions

The resistive switching characteristics of the device were evaluated under DC programming conditions. The typical current–voltage (*I–V*) characteristic of the Ta/TaO_x_/Al_2_O_3_/Pd device under DC sweep mode from −6 V to 6 V is shown in Figure 1d. The device is forming-free, and no abrupt change of current in both SET and RESET switching processes is observed, indicating a bipolar analog resistive switching feature. Within 6 V and −6 V stop voltages on SET and RESET processes, a ~10^3^ ratio between HRS and LRS can be obtained (read voltage is 1 V), which is larger than our recent work of similar TaO_x_/Al_2_O_3_ stack device (~10^2^ ratio, Ti/AlO_x_/TaO_x_/Pt) [34].

**Figure 2:**
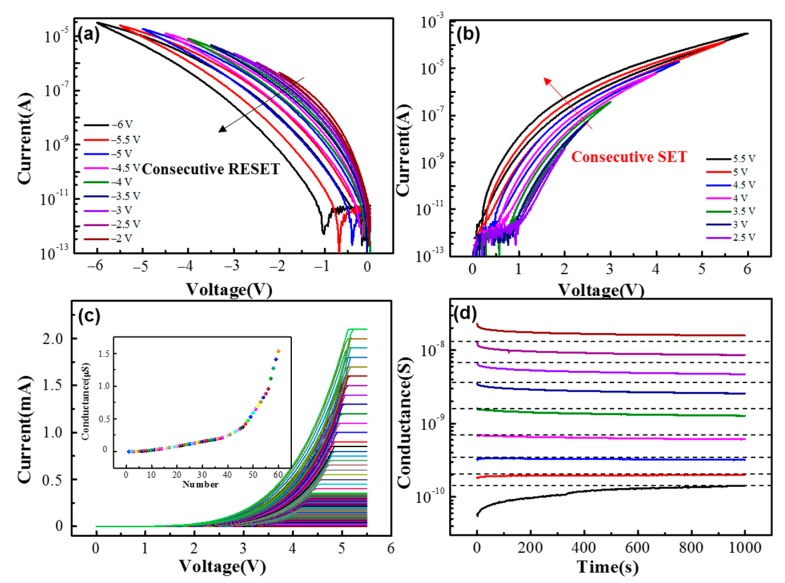
Bipolar analog-resistive switching characteristics of the Ta/TaO_x_/Al_2_O_3_/Pd device under the DC sweeping mode: (**a**) consecutive DC sweeping with different stop voltages from −2 to −6 V in the RESET process; (**b**) consecutive DC sweeping with different stop voltages from 2.5 to 5.5 V in the SET process; (**c**) consecutive DC sweeping with different compliance currents from 500 nA to 2.2 mA in the SET process (inset: 60 different conductance states obtained by modulating different compliance currents); and (**d**) retention characteristics of nine different resistance states of the Ta/TaO_x_/Al_2_O_3_/Pd device.

To further demonstrate the analog characteristics, the DC sweep with different working voltages and without compliance currents (SET voltage: 2.5 V, 3 V to 5.5 V; and RESET voltage: −2 V, −2.5 to −6 V) and the DC sweep with different compliance currents during SET process are shown in Figure 2. The initial resistance of the device is ~10^11^ Ω (read at 1 V). When the positive sweeping voltage is applied to the device, the resistance of the device is retained until the voltage reaches 2.5 V, then the resistance gradually decreases. During the consecutive SET process, as shown in an inset of Figure 2b, the responding currents (read at 1 V) can gradually increase with the increment of the stop voltages, indicating that different conductance states can be obtained in the SET process. Various conductance states can also be obtained by setting different compliance currents during the SET process. With compliance currents from 500 nA to 2.2 mA, the corresponding *I–V* curves and the 60 different resulting conductance states are shown in Figure 2c and the inset, respectively. The RESET process can be implemented by applying a negative DC sweeping voltage to the device. As shown in Figure 2a, eight consecutive negative DC sweeps with various stop voltages are applied to the device. As the voltages decrease from −2 to −6 V with a −0.5 V step, the device is switched to a higher resistance state after each step. Moreover, the multilevel resistance states can be preserved within satisfying retention time, as shown in Figure 2d. The multilevel resistance states are obtained by consecutive positive voltage sweepings (2 to 6 V with a 0.25 V step). After each sweeping, the device resistance states are monitored by a series of 1 V reading pulses at 0.5 s intervals. As it is shown in Figure 2d, though with slightly decay, nine different states can be clearly distinguished after 1000 s. 

**Figure 3:**
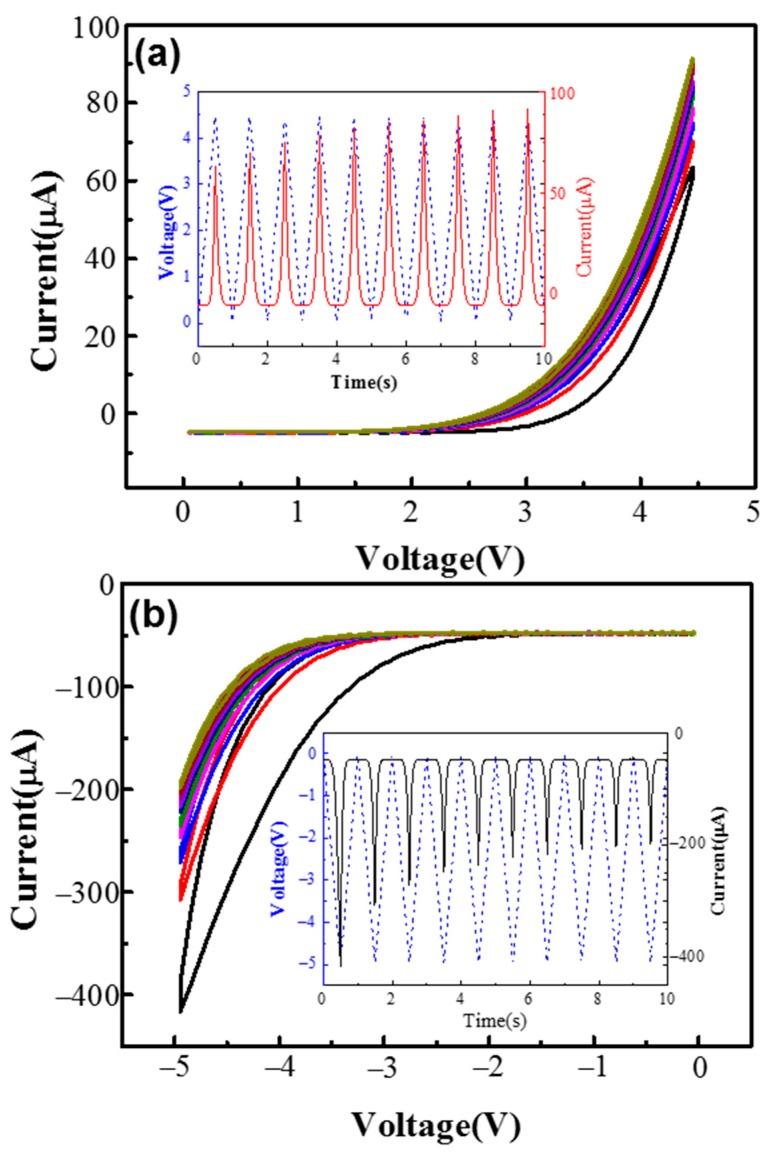
(**a**) Gradual SET under positive triangle pulses (from 0 to 4.5 V) (inset: the *I-t* and *V-t* curves of (**a**), representing the gradual increasing of current with time); (**b**) gradual RESET under negative triangle pulses (from 0 to −5 V) (inset: the *I-t* and *V-t* curves of (**b**), representing the gradual decreasing of current with time).

The characteristics of the bipolar analog-resistive switching in pulse mode are investigated via positive (0 to 4.5 V) and negative (0 to −5 V) triangle pulses, as shown in Figure 3a,b, respectively. The curves of current and voltage versus time for the SET and RESET processes are shown in the insets of Figure 3a,b, respectively. These results further confirm the analog resistive switching characteristics under both positive and negative pulses. The results reveal that in both the SET and RESET processes, gradual tuning of the multilevel conductance states can be obtained. Bipolar analog resistive switching characteristics are fully analogous to the biology synapse; thus, the devices have the potential to mimic synaptic functions in neuromorphic computing system.

**Figure 4:**
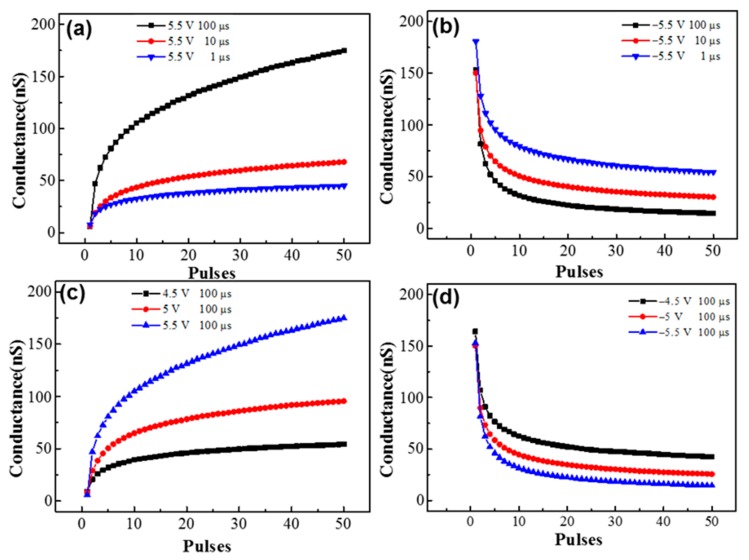
The measured long-term potentiation and long-term depression synaptic function with identical pulses. A total of 50 pulses with 5.5 V pulse amplitude and different pulse widths for (**a**) potentiation (1 μs, 10 μs, and 100 μs); (**b**) depression (1 μs, 10 μs, and 100 μs); 50 pulses with 100 μs pulse width and a different pulse amplitude (**c**) for potentiation (4.5 V, 5 V, and 5.5 V) and (**d**) for depression (−4.5 V, −5 V, and −5.5 V).

Long-term potentiation/depression (LTP/LTD) is when the synaptic weight can be changed gradually under spiking signals and the changed weight can be maintained from several minutes to years [35]. To evaluate the long-term potentiation/depression of a device, 50 consecutive pulses with different pulse amplitudes and widths are applied to the device, as shown in Figure 4. All the conductance of the device is monitored by 1 V reading voltage. The change of conductance can be modulated by different amplitudes and widths. As shown in Figure 4a,b, the amplitude here was fixed at 5.5 V during potentiation and −5.5 V during depression, with different widths (1 μs, 10 μs, and 100 μs). In addition, Figure 4c,d show the potentiation and depression with a fixed 100 μs width and different amplitudes (potentiation: from 4.5 to 5.5 V; depression: from −4.5 to −5.5 V). With a higher amplitude or larger width, the change of the conductance is increased in both potentiation and depression. For our device, when the pulse amplitude (write voltage) is ~±4.5 V and the pulse width is 1 μs, the write current is around ~10^−5^ A; thus, the switching energy is 50 pJ per spike. To conclude, the device conductance is continuously increased by positive pulses, which can mimic long-term potentiation. In addition, the device conductance is continuously decreased by negative pulses, which can mimic long-term depression. 

**Figure 5:**
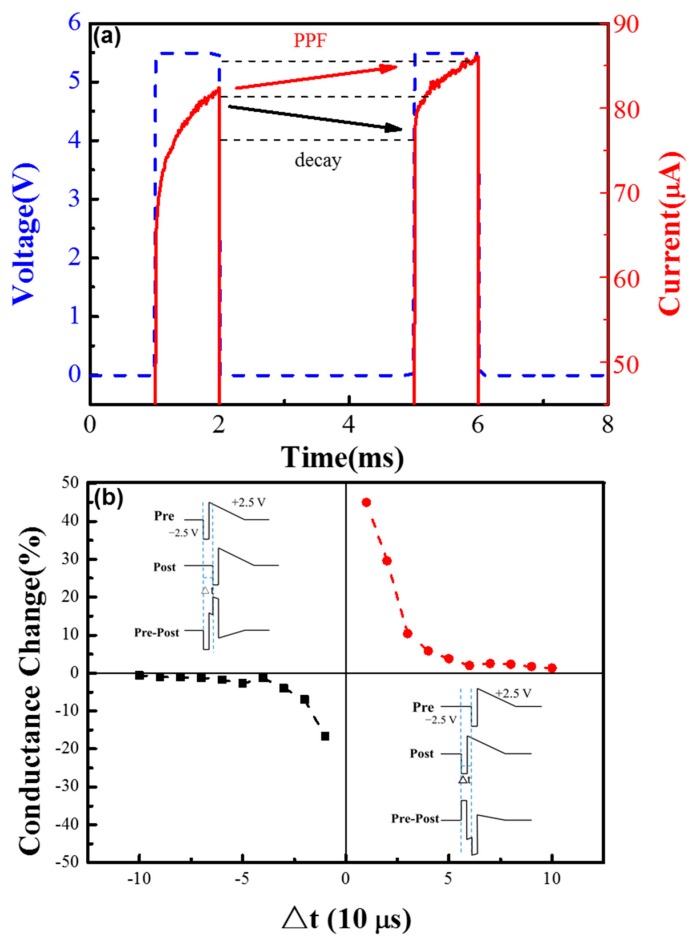
(**a**) The short-term plasticity: paired-pulse facilitation (PPF) synaptic function of the Ta/TaO_x_/Al_2_O_3_/Pd device; (**b**) illustration of spike signals and spiking-time-dependent plasticity (STDP) function of the device. The individual pre-synaptic or post-synaptic spike signal is designed as a pair of pulses (−2.5 V, 10 μs pulse and 2.5 V triangle pulse) applied to the top and bottom electrode, respectively.

Moreover, the device can emulate other synaptic features, such as paired-pulse facilitation (PPF) and spiking-time-dependent plasticity (STDP), as shown in Figure 5. Most research on artificial synapses focuses on the long-term plasticity, because long-term changes provide a physiological substrate for learning and memory. However, short-term plasticity is also significant, since it supports a variety of computations, such as synaptic filtering, adaptation, and enhancement of transients, decorrelation, burst detection, and sound localization [36]. PPF is an important kind of short-term plasticity. In biological synapses, PPF functions can be described as follows: the second post-synaptic response current becomes larger than the first under two successive spike stimuli, with the interval time of spikes less than recovery time [8]. The experimental demonstration of PPF functions in our device is shown in Figure 5a. When a pair of pulses is applied to the device, the conductance gradually increases during the positive pulses, and the maximum responding current of the second pulse is clearly larger than the first, and a decay phenomenon can be observed during the pulse interval, which is similar to the PPF in the biological system.

In biological systems, synaptic weight can be modulated by the temporal relationship of the activity between the pre- and post-synaptic neurons, which is called spiking-time-dependent plasticity (STDP). According to STDP, the change of synaptic weight (ΔW) is a function of the time difference between pre- and post-synaptic activity (Δt). To emulate the STDP function in the device, a pair of pulses acting as the spiking signals with different time intervals is applied to the device. Individual pre-synaptic or post-synaptic spiking signals are designed as a pair of pulses (−2.5 V, 10 μs pulse and a 2.5 V triangle pulse) applied to the top and bottom electrode, respectively, as shown in Figure 5b. It should be noted that an individual positive signal or an individual negative signal is not strong enough to modulate the resistance of the device. As shown in Figure 5b, the effective signal to the device is the pre-synaptic signal minus the post-synaptic signal. When the pre-spike appears before the post-spike (Δt > 0), the conductance (synaptic weight) of the device is enhanced (potentiation), and the change in weight decreases with the increase of Δt. On the contrary, when the pre-spike appears after the post-spike, the conductance of the device depresses and the change of the weight decreases with the increase of Δt. The measurement result shows that the Ta/TaO_x_/Al_2_O_3_/Pd device can emulate the STDP learning rules successfully, which has potential to be used in the spiking neuron network (SNN).

**Figure 6:**
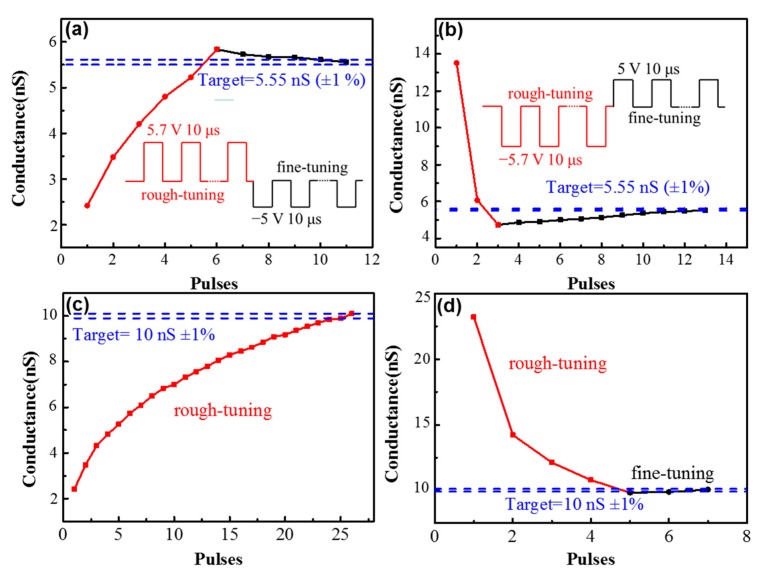
The bipolar conductance tuning to randomly determined target states (5.5 nS ± 1% and 10 nS ± 1%) under pulses with different initial states: (**a**) the initial state is 2.41 nS, and the target conductance state is obtained by 5.7 V positive pulses for rough-tuning and −5 V negative pulses for fine-tuning; (**b**) the initial state is 13.5 nS, and the target conductance state is obtained by −5.7 V negative pulses for rough-tuning and 5 V positive pulses for fine-tuning; (**c**) the initial state is 2.2 nS, and the target state is obtained only by rough-tuning; and (**d**) the initial state is 23 nS, and the target state is obtained by rough-tuning and fine-tuning methods.

**Figure 7:**
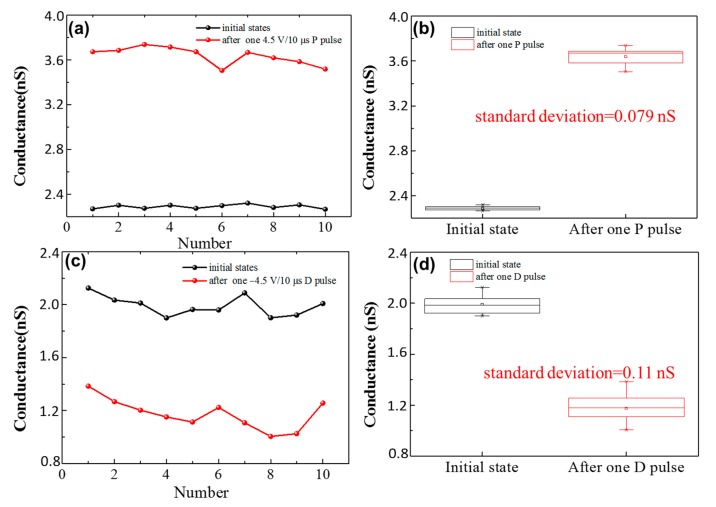
(**a**) The conductance states after one 5 V/10 μs P pulse from nearly the same initial states. (**b**) Conductance distribution from (a), where the standard deviation is 0.079 nS. (**c**) The conductance states after one −5 V/10 μs P pulse from nearly the same initial states. (**d**) Conductance distribution from (c), where the standard deviation is 0.11 nS.

To fully explore bipolar conductance tuning characteristics and demonstrate the potential application of the device in some specific neuromorphic computing systems like ANN, determined target conductance states with different initial states have been tested. As shown in Figure 6a, the initial state is 2.41 nS, after two tuning processes: 5.7 V positive pulses with 10 μs width for rough-tuning, and −5 V negative pulses with 10 μs width for fine-tuning. The target conductance state of 5.5 nS can be obtained with little deviation (<1%). The same target conductance state can also be obtained when the initial conductance state is 13.5 nS, by −5.7 V negative pulses with 10 μs width for rough-tuning and 5 V positive pulses with 10 μs width for fine-tuning, as shown in Figure 6b. As shown in Figure 6c,d, another target conductance state (10 nS), can be obtained with little deviation. It is worth noting that the target conductance states are determined randomly. Based on this result, it can be proven that precision is achieved across a wide dynamic range. Writing error is a standard plot when characterizing resistive switching write noise. The write error of the device has been tested, as shown in Figure 7. A DC sweeping with 100 μA compliance current is used to get nearly the same initial states. Only one programming pulse (4.5 V/10 μs for potentiation and −4.5 V/10 μs for depression) is applied after each DC sweeping. The conductance states (total 10 cycles) are obtained by 1 V reading voltage. As shown in Figure 7b,d the standard deviation is 0.079 nS after one potentiation pulse, and 0.11 nS after one depression pulse, respectively. The dynamic range is around 20 nS under 4.5 V/10 μs training pulses. As a result, the write error is only around 0.6% of the total dynamic range.

**Figure 8:**
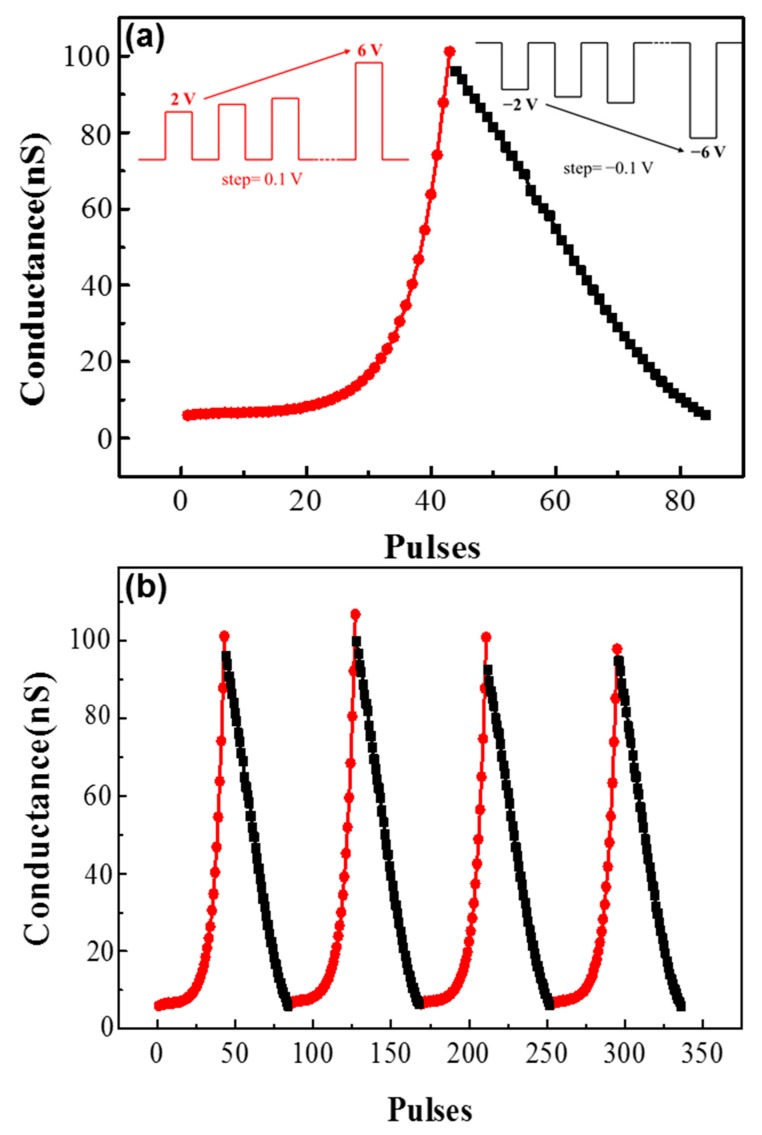
The measured long-term potentiation/depression synaptic function with non-identical training pulses: (**a**) the training pulses with increasing amplitudes (potentiation: from 2 to 6 V, 100 μs; depression: from −2 to −6 V, 100 μs); and (**b**) the weight updates, recorded in four training cycles.

The recognition accuracy of the ANN highly depended on the linearity of the synaptic weight change—i.e., the recognition accuracy is low under high non-linearity [37,38]. However, as shown in Figure 4, the device is highly non-linear. To improve the linearity of the conductance change of the device, a non-identical pulse scheme is adopted, as shown in Figure 8. The training pulses are fixed at width but with increasing amplitudes. The amplitude range of the potentiation process is from 2 to 6 V with 0.1 V steps, and the range of the depression process is from −2 to −6 V with −0.1 V steps. The weight updates are recorded in four training cycles, as shown in Figure 8. The non-linearity factor (NL) has been calculated by NL = average ($\frac{G-G_{linear}}{G_{linear}}$) [39], so the non-linearity factors of the normal training method are 1.09, 1.427, and 1.332 respectively, based on the data in Figure 4a. In addition, the non-linearity factors of the incremental training method are −0.62 for long-term potentiation and 0.13 for long-term depression, based on the data in Figure 8a. 

**Figure 9:**
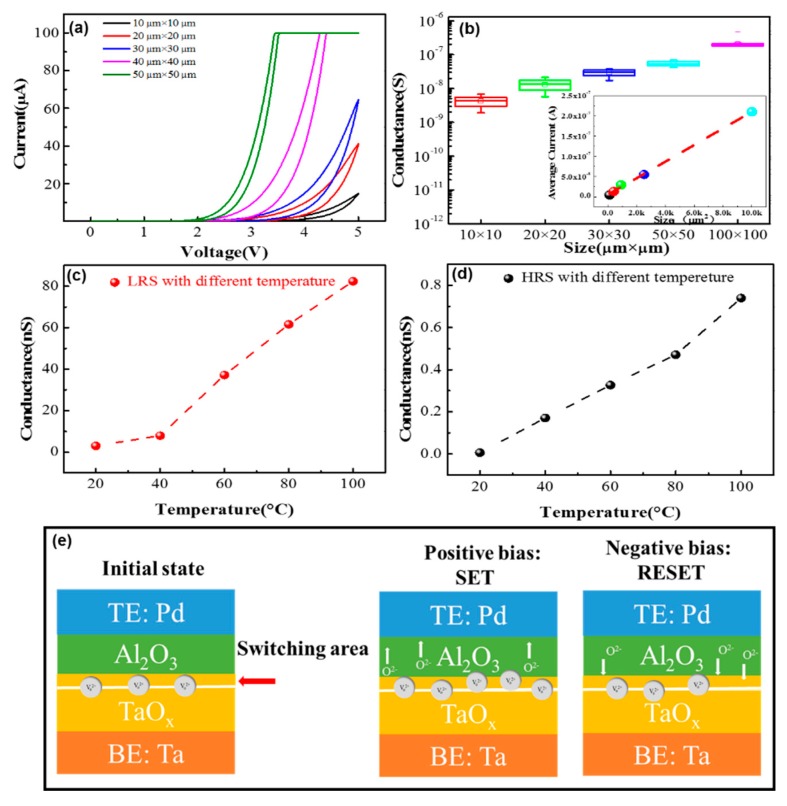
(**a**) The *I–V* curves of the first SET process with different electrode area sizes (from 10 μm × 10 μm to 100 μm × 100 μm). (**b**) Conductance distribution at LRS with different sized areas (the conductance states are obtained by 1 V reading voltage in 25 different devices) (inset: the linear fit result confirms that the device conductance scales linearly with device areas). (**c**) Conductance of LRS with different temperatures from 20 to 100 °C; (**d**) Conductance of HRS with different temperatures from 20 to 100 °C. (**e**) The schematic of the switching mechanism of the device; the switching area is the interface of TaO_x_-Al_2_O_3_, and the push-and-pull of the oxygen ions in the surface can change the resistance of the interface layer.

The investigation of the switching mechanism of the device is shown in Figure 9. The conductance of the filamentary memristors mostly depends on the size and morphology of the conductive filament with several nanometers diameter in the device. Thus, the conductance of filamentary memristor does not significantly change with the change of the electrode areas. The *I–V* curves of the 1st SET process and conductance distribution of 25 different devices at LRS with various electrode areas (from 10 to 100 μm^2^) are shown in Figure 9a,b, respectively. In Figure 9a, the current level after SET shows a positively proportional relationship with the electrode area. In statistical analysis of 25 devices at LRS in Figure 9b, such a trend can be more clearly seen in the plotting of the conductance with the electrode area. As shown in inset of Figure 9b, the linear fit result confirms that the device conductance scales linearly with the device area. As a result, the switching occurs across the entire electrode area, but not just within a local filament, suggesting a non-filamentary switching mechanism of Ta/TaO_x_/Al_2_O_3_/Pd device. The temperature dependencies of the device conductance at LRS and HRS are studied in Figure 9c,d, respectively. With the increase of temperature, the conductance at both LRS and HRS increases as well, indicating the semiconductor conduction behavior of the device. To explain the switching mechanism of the device, we proposed a simple model [40], shown in Figure 9e. The device can be divided into three parts: a barrier layer (Al_2_O_3_), a switching area (interface of Al_2_O_3_ and TaO_x_), and a conductive oxidation layer (TaO_x_). The switching area is located at the interface of Al_2_O_3_ and TaO_x_. During SET operation, a positive voltage is applied on the top electrode, the oxygen ions in the barrier layer are pulled away from the interface layer, and the materials in the interface are reduced. During RESET operation, a negative voltage is applied on the top electrode, the oxygen ions in the barrier layer are pushed into the interface layer, and the materials in the interface are oxidized. The push-and-pull of the oxygen ions in the surface can change the resistance of the device.

To implement neuromorphic computing, the device should be integrated into an array. To operate an array, a half-bias scheme is a common method. However, our device has a low ON/OFF ratio (<100) between the selected voltage and the half-selected voltage, which may cause a sneak path issue during write operation. As a result, it is hard for our device to implement a dense crossbar array without the help of a transistor or selector device. A one transistor one resistor (1T1R) or one selector one resistor (1S1R) structure should be adopted to overcome the sneak path issue during writing operation. The device structure can still be optimized to improve the linearity of the conductance changes and decrease the working voltage. In addition, the detailed non-filamentary switching mechanism in this device needs to be further explored.

## 4. Conclusions

In this paper, a Ta/TaO_x_/Al_2_O_3_/Pd memristor is fabricated, to be used as artificial synapse. The device shows bipolar analog-resistive switching behavior. Moreover, multilevel conductance states with a satisfying retention time (>1000 s) can be obtained by modulating voltages or compliance currents under DC sweeping mode. Based on the bipolar analog switching, synaptic functions, including long-term potentiation/depression, paired-pulse facilitation, and spiking time dependent plasticity are successfully mimicked. For ANN applications, the determined target conductance, the linearity, and the writing errors are carefully examined. The results suggest that as an artificial synapse, the Ta/TaO_x_/Al_2_O_3_/Pd memristor is a promising candidate for neuromorphic computing.
